# Quality of life in Arab women with breast cancer: a review of the literature

**DOI:** 10.1186/s12955-016-0468-9

**Published:** 2016-04-27

**Authors:** Bouchra Haddou Rahou, Karima El Rhazi, Fatima Ouasmani, Chakib Nejjari, Rachid Bekkali, Ali Montazeri, Abdelhalem Mesfioui

**Affiliations:** Laboratory of Genetic, Neuroendocrinology and Biotechnology, University Ibn Tofail, Faculty of Sciences, Kenitra, Morocco; Department of Epidemiology and Public Health, Faculty of Medicine and pharmacy of Fez, Sidi Mohamed Ben Abdillah University, Sidi Mohamed Ben Abdillah, Morocco; Fondation Lalla Salma Prevention and Treatment of Cancers, Rabat, Morocco; Mental Health Research Group, Health Metrics Research Centre, Iranian Institute for Health Sciences Research, ACECR, Tehran, Iran

**Keywords:** Quality of life, Breast cancer, Arab women

## Abstract

**Background:**

Quality of life has become an important concept in cancer care. Among the quality of lifestudies in cancer patients, breast cancer has received most attention. This review reports on quality of life in Arab patients with breast cancer.

**Methods:**

The search was conducted using inclusion and exclusion criteria and in accordance with Preferred Reporting Items for Systematic Reviews and Meta-Analyses (PRISMA). The databases consulted were PubMed, Sciences Direct, Index Medicus for Wordl Health Organization Eastern Mediterranean, African Journals Online and African Index Medicus.

**Results:**

Thirteen articles from eight countries met the inclusion criteria. The EORTC quality of life questionnaires (QLQ-C30 and QLQ-BR23) were the most used instrument (7 out of 13). The results showed that good scores of global health were recorded at Arab women living in United Arab Emirates (mean score = 74.6) compared to other countries. The results indicated that there was a difference in quality of life scores and its associated factors among Arab women with breast cancer.

**Conclusion:**

This paper is the first that reviewed published research on quality of life among Arab women with breast cancer. We found that insufficient results-related information is available.

**Electronic supplementary material:**

The online version of this article (doi:10.1186/s12955-016-0468-9) contains supplementary material, which is available to authorized users.

## Background

Quality of life (QOL) has become an important outcome measure in the treatment of cancer patients during the last decade. It is a multidimensional construct encompassing perceptions of both positive and negative aspects of dimensions such as physical, emotional, social and cognitive functions, as well as the negative aspects of somatic discomfort and other symptoms produced by a disease or its treatment [1]. Clinical trials have shown that changes in QOL are associated with changes in clinical variables including survival [2].

It has been shown that assessing QOL in cancer patients could contribute to improve treatment and could be an important prognostic factor [3–6].

Among the QOL studies in cancer patients, breast cancer has received most attention. This is partly due to the increasing number of patients. Statistics show that breast cancer is by far the most frequent cancer among women in the world, with an estimated 1,67 million new cases diagnosed in 2012 [7]. On the other hand, through early detection programs and more effective treatments, more women with breast cancer are surviving longer [8].

However, the physical, functional, psychological and social difficulties of the women treated for cancer can compromise their QOL. The QOL studies in breast cancer patients reported that anxiety, depression, pain, fatigue, and arm morbidity were the most reported symptoms [9].

Patients receiving chemotherapy might experience several side-effects and symptoms that negatively affect their QOL [9] and patients who underwent mastectomy indicated lower body image and sexual functioning than those who did not [9].

Many psychosocial and medical factors, like age, patient education, spousal support and employment status, financial stability, disease stage, have been reported in the literature to predict the QOL of patients [1, 10].

The Arab world has a total of 22 countries spread across Northern Africa and Western Asia, including the Middle East. Data from Arab countries on breast cancer vary according to region and country. In Arab countries, the breast cancer represents 14 % to 42 % of all female cancers [11]. Age-adjusted standardized incidence rates (ASR) were reported to vary from 9.5 to 50 cases per 100,000 women per year [11]. 50 % of cases are younger than 50 years compared to 25 % in developed countries [11]. Cancer remains a taboo in most Arab countries [11]. The majority of people still refer to it as “other disease” and remain afraid of mentioning it by name [11]. Arab womenshare a set of different cultural, norms and beliefs and studies have shown that patient-based outcomes could be affected by cultural experiences and ethnic backgrounds [12, 13].

Analysis of a large international database of the European Organization for Research and Treatment in Cancer Quality of Life Questionnaire (EORTCQLQ–C30) indicated that,compared with patients from the United Kingdom; physical and social functioning were less important in predicting the global QOL of patients from Islamic countries, while cognitive functioning was more influential for South Asia and Latin America [14].

Recently, healthcare providers in Arab countries have started looking at the QOL of women diagnosed and living with breast cancer and the QOL is receiving more and more attention from researchers. The aim of the present review is to collect and examine publication that have appeared in English and French biomedical journals. The findings will constitute an evidence base for the cancer control programs in Arab countries and may reveal findings that will ultimately require a specific approaches for assisting Arab patients according to the specificities of their culture and religion.

## Methods

### Search strategy

The search was conducted to identify studies reporting the QOL in Arab women with breast cancer in accordance with Preferred Reporting Items for Systematic Reviews and Meta-Analyses statement criteria (PRISMA) [15].

A systematic literature search was conducted using PubMed, Sciences direct,Index Medicus for World Health Organization (WHO) Eastern Mediterranean, African Journals Online and African Index Medicus (AIM). The reference lists of all identified publications were checked to retrieve other relevant publications, which were not identified through of the computerised search. until May 2015, without restrictions of language or year of publication.

We use the term *quality of life* to be synonymous with the expression *health-related quality of life*.

The search strategy included the following keywords or their combinations in titles of publications:*quality of life, health related quality of life, breast cancer, breast carcinoma, North Africa, Middle East, Arabic countries, Morocco, Algeria, Tunisia, Libya, Mauritania, Oman, Saudi Arabia, Bahrain, Comoros, Djibouti, Egypt, United Arab Emirates, Iraq, Jordan, Kuwait, Lebanon, Palestine, Qatar, Somalia, Sudan, Syria, Yemen.*

### Inclusion and exclusion criteria

Studies were included if they described aspects of the quality of life or health quality of life in Arab women with breast cancer, and presented the results on the quality of life evencross- cultural adaptation studies.

We excluded studies that did not pertain Arab women or reported on all types of cancers and did not specify the breast cancer. Furthermore, we excluded studies and information published only as abstracts.

### Type of studies

Were included both qualitative and quantitative studies in this review. For the quantitative studies, both observational and interventional were included.

### Analyzed outcomes

The analyzed outcomes were global QOL score, QOL domains, factors associated with QOL, perceptions and attitudes.

## Results

**Fig. 1:**
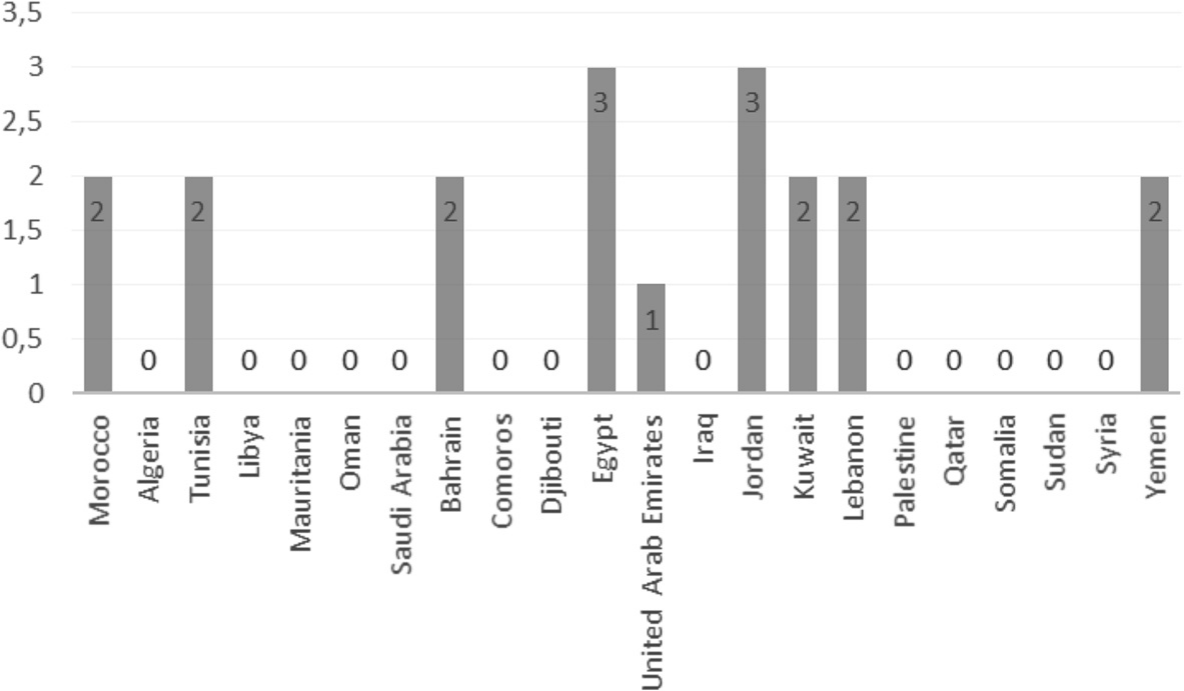
Results of the literature search made for each Arabic country

Our findings indicate that studies on QOL in Arab women with breast cancer are very scarce. Indeed, among the 22 Arab countries, only 09 have conducted studies in this field (Fig. 1).

**Fig. 2:**
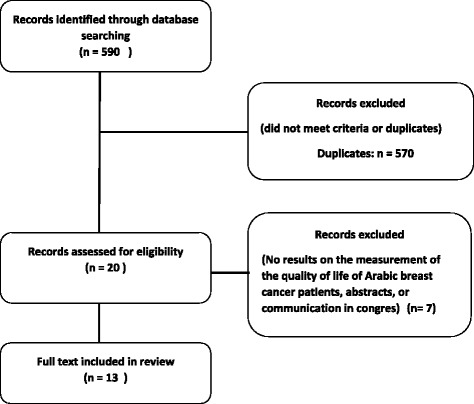
Flow chart of process of systematic literature search in accordance with PRISMA

Figure 2 shows the flowchart of the systematic review process followed. The initial search identified a total of 590papers; among them 570 manuscriptswere excluded due to duplication. Twenty potential articles were identified based on the relevance of the abstracts. Followinga thorough review of the full text,thirteen articles were eligible for inclusion.

### Characteristics of included studies

Thirteen studies met the inclusion criteria. All were published between 1997 and 2014. The first study was conducted in 1997 while the second until 2008. The other eleven studies all published in the last six years. The quality assessment of selected studies are shown in Table 1.

**Table 1:** Quality assessment of included studies on “Quality of life of Arabic women with breast cancer”

Author/Reference	Relevant to this SR	Aims clearly stated	Appropriate study method	Sample representative of target population	Confounding and bias considered	Good response rate?	Were questions piloted/validated?	Tables/figures understandable	Can results be applied to local situation?	Accepted as Type IV evidence?
El Sharkawi et al. [17]	Yes	Yes	Yes	No	Yes	Yes	Yes	Yes	Yes	Yes
Awad et al. [18]	Yes	Yes	Yes	No	Yes	Yes	Yes	Yes	Yes (just for breast cancer patients)	Yes
Alawadi and Ohaeri [19]	Yes	Yes	Yes	No	Yes	Yes	Yes	Yes	Yes	Yes
Masmoudi et al. [20]	Yes	Yes	Yes	No	Yes	Yes	Yes	Yes	Yes	Yes
Mostafa et al. [21]	Yes	Yes	Yes	No	ND	Yes	Yes	Yes	Yes	No (type II)
Al-Naggar et al. [22]	Yes	Yes	Yes	No	Yes	Yes	Yes	Yes	Yes	Yes
Huijer and Abboud [23]	Yes	Yes	Yes	No	Yes	Yes	Yes	Yes	Yes	Yes
Denewer et al. [24]	Yes	Yes	Yes	No	ND	Yes	Yes	Yes	Yes	Yes
Ba-Khubaira and Al-Kahiry [25]	Yes	Yes	Yes	No	Yes	Yes	Yes	Yes	Yes	Yes
Jassim and Whitford [26]	Yes	Yes	Yes	Yes	Yes	Yes	Yes	Yes	Yes	Yes
Jassim and Whitford [16]	Yes	Yes	Yes	No	Yes	Yes	ND (qualitative study)	Yes	Yes	Yes
El Fakir et al. [27]	Yes	Yes	Yes	No	Yes	Yes	Yes	Yes	Yes (not for other regional languages)	Yes
Abu-Helalah et al. [28]	Yes	No	Yes	No	Yes	Yes	Yes	Yes	Yes	Yes

A total of **4132** Arab women with breast cancer were studied in these investigations. Characteristics of the studies are shown in Table 2.

**Table 2:** Characteristics of included studies

Author/Year/Reference	Country and setting	Study design and Population	Main focus	QOL Assessment	Outcome	Comparison	Main finding and effects
El Sharkawi et al. [17]	Egypt Surgery and clinical and nulear Medicine departments, Alexandria Men University Hospital	Cross sectional study 272 women with early Breast cancer who are under treatment	Determine the effects of the treatment on the QOL of Egyptian women with early Breast Cancer	Linear analogue self-assessment (LASA) scales	the four domains of QOL of women having adjuvant therapy were significantly altered compared to those who underwent mastectomy alone triple modality adversely affected global QL the most compared to radiotherapy or chemotherapy radiotherapy had significantly less effect on QL compared to chemotherapy triple modality predicted the worst QOL	Patients were divided into four groups: 1. mastectomy alone, 2. surgery plus radiotherapy, 3. surgery plus chemotherapy 4. triple modality	Quality of life measures should be incorporated in the evaluation of treatment Patients should receive health education on the effects of each therapy
Awad et al. [18]	United Arab Emirates The Breast Cancer Clinic at Tawam Hospital, Al Ain	Cross sectional study 87 women with breast cancer and who are of Arabic origin 3 months after surgical treatment	Assess the psychometric properties of the Arabic version of the EORTC QLQ-C30 and QLQ-BR23 in Arab breast cancer patients	The Arabic version of EORTC QLQ-C30 and QLQ-BR23	Participants had a mean score for global QOL of 74.6 The QLQ-C30 discriminated between mastectomy and lumpectomy patients on the emotional and cognitive function scales and QLQ-BR23 discriminated as well on the function scales and for systemic side effects.	Group I :mastectomy patients Group II:lumpectomy patients	Patients’ perceptions extend beyond the negative physical and functional impact of cancer to the individuals’ perceptions of their general well-being.
Alawadi and Ohaeri [19]	Kuwait The medical oncology department of the Kuwait Cancer Control Center	Cross-sectional a comparative study using the EORTC Quality of Life Questionnaire 348 women with breast cancer receiving chemotherapy	Highlight the HRQOL scale scores for Kuwaiti women in stable clinical condition with breast cancer, in comparison with the international data. Assess the socio-demographic and clinical variables that predict the five functional scales and global QOL (GQOL) scale of the QLQ – C30	The Arabic version of EORTC QLQ-C30 and QLQ-BR23	The mean score of global QOL scale (GQOL) was 45.3 The patients had poor to average functioning and intense symptom experience. Younger women had poorer HRQOL scores The significant associations of disease stage with role functioning, diarrhea and future perspective In regression analysis, social functioning accounted for the highest proportion of variance for GQOL.	**-**	The biological and treatment side effect factors seemed to be more important than family and institutional supports A longitudinal study is needed to confirm this trend.
Masmoudi et al. [20]	Tunisia Department of Medical Oncology in Sfax University Hospital	Descriptive study 23 women with early breast cancer receiving adjuvant chemotherapy	Assess the feasibility of QOL assessment in a cohort of Tunisian cancer patients	The Arabic version of EORTC QLQ-C30	Participants had a mean score for global QOL of 72.5 pre chemo And 68.5 during chemo. A significant deterioration in physical, cognitive, and social functioning, between the pre-treatment and on-treatment assessments	Group I:pre-treatment Group II:on-treatment	improvement of cancer care infrastructure and public education is still needed before reliable QOL studies can be performed
Mostafa et al. [21]	Egypt El- Minia oncology center	Interventional hospital based study, 180 female breast cancer patients recei-ving treatment	Assess QOL, its relation to different variables related to cancer Trial to improve the QOL of patients and their families through communication, counseling, restorative (rehabilitative) therapy, social and medical support.	The Arabic version of EORTC QLQ-C30 and QLQ-BR23	38.3 % of studied females had poor global health status/QOL 52.8 % had good global health status/QOL There is a significant change in physical, role, emotional, cognitive and social functioning in pre- and post-intervention assessment.	180 female breast cancer patients in pre-intervention 75 patients (with global health status/QOL score value of ≤ 50) in post intervention phase	Need to provide comprehensive care for breast cancer survivors
Al-Naggar et al. [22]	Yemen The outpatient of National Oncology Centre (NOC), Sana’a	Cross-sectional study 106 female breast cancer patients underwent treatment.	Determine the QOL among breast cancer patients in Yemen based on socio-demographic and clinical characteristics	Functional Assessment of Cancer Treatment-Breast (FACT-B) questionnaire	Years after diagnosis, family monthly income and radiotherapy were significantly associated with total QOL of the breast cancer patients	-	Age, occupation, family history of breast cancer, size of tumor, chemotherapy and tamoxifen were not significantly influence QOL
Huijer and Abboud [23]	Lebanon American University of Beirut-Medical Center (AUB-MC)	Cross-sectional descriptive survey 200 Lebanese adult patients with cancer including 89 women with breast cancer who are diagnosed for more than one month	Evaluate the QOL, symptom prevalence and management, functional ability, and quality of care provided to Lebanese women with BC	The Arabic version EORTC-QLQ-C30	Mean score for global QOL: 59.64 High scores were reported on functional ability, medical care, spirituality, and relationships. Factors significantly associated with QOL: Payments per month for medical expenses, presence of metastasis, time since diagnosis, symptoms, and type of treatment received .	-	The impact of clinical characteristics on QOL is far more significant than demographic characteristics
Denewer et al. [24]	Egypt the Oncology Center–Mansoura University	Prospective study 200 Egyptian women within 2 months–2 years from their primary surgery	Evaluate QOL, body image, and patient satisfaction comparing between traditional mastectomy alone and sparing mastectomy with immediate autologous breast reconstruction	Breast impact of treatment scale (BITS) Body satisfaction scale (BSS)	Patient with breast reconstruction had a high mean score of BSS: 14.44 out of total degrees of 20 No difference was found between the two groups as regard the BITS score	Group I :patients underwent sparing mastectomy with immediate autologous breast reconstruction Group II:100 Patients underwent traditional mastectomy	Egyptian women with breast cancer show better QOL and body image satisfaction outcomes following immediate breast reconstruction.
Ba-Khubaira and Al-Kahiry [25]	Yemen Central Public Health Laboratories – Aden branch	Cross-sectional study 58 Yemeni patients with early stage breast cancer. During follow-up for the last 2 years after they finished treatment**.**	Determine the QOL of Yemeni patients in Aden after treatment of early stage breast cancer below 50 years compared to 50 years and more of age.	FACT-B Questionnaire	The overall QOL reported in this study among breast cancer Yemeni patients in Aden was 77.6 and the breast cancer-specific subscale (BCS) was 21.2. Yemeni patients with early breast cancer are having lower QOL after treatment; this QOL was deteriorated among younger patients when compared to older patients.	Group I: early stage breast cancer below 50 years Group II: 50 years and more of age.	-Evaluation of the post-treatment QOL of cancer patients should be a part of the evaluation criteria of cancer therapy in Aden.
Jassim and Whitford [26]	Bahrain The main governmental Hospital, Salmaniya Medical Complex	Descriptive cross sectional study 239 Bahraini breast cancer survivors	Describe the QOL of Bahraini women with breast cancer and its association with their sociodemographic and clinical data.	The Arabic version of EORTC-QLQ-C30	Participants had a mean score for global QOL of 63.9 Average to good QOL functioning and low to average symptoms experience Factors associated to lower QOL: marital status, menopausal status, metastases, monthly income and type of surgery.	-	Bahraini BC survivors reported favorable overall global quality of life. Special care should be given to women with cancer related symptoms and metastatic lesions
Jassim and Whitford [16]	Bahrain Oncology Center in Salmaniya Medical Complex	Qualitative study 12 Bahraini women diagnosed with breast cancer. Who were deemed to have coped to differing degrees both during and following their initial diagnosis.	Explore the Experiences, beliefs, perceptions and attitudes of Bahraini women with breast cancer towards their quality of life.		Global QOL was expressed in terms of being able to perform every day chores and the ability to function in one’s daily role as a woman, wife, daughter and employee Hair loss was a major side effect of treatment the important role played by the family and husband in treatment decisions the use of traditional clothing (hijab and abaya) to hide hair and body changes the importance of spirituality and religion to cope with the disease	-	The finding aid healthcare professionals in planning appropriate interventions that meet the patients’ needs
El Fakir et al. [27]	Morocco National institute of oncology in Rabat and oncology center of Ibn Rochd hospital in Casablanca	Cross-cultural adaptation of the EORTC QLQ-BR23 105 women with breast cancer	Assess the reliability and validity of this translation for use in Morocco.	Moroccan Arabic version of the EORTC QLQ-BR23	Scores for different scales ranged from 34.0 to 77.8. Body image had the higher score (83.33) Systemic therapy side effects” scale had the lower score (median 57.14)	-	QLQ-BR23 questionnaire could be used in clinical trials that evaluate the impact of specific interventions on the QOL of Moroccan patients with breast cancer
Abu-Helalah et al. [28]	Jordan the Radiation Oncology Department at Al-Bashir	Cross-sectional study 236 Jordanian breast cancer survivors	Obtain such data Quality of Life and Psychological Well-Being of Breast Cancer Survivors in Jordan Assess predictors with calculated scores	EORTC QLQ-C30, the Breast Module QLQ-BR23 the Hospital Anxiety and Depression Scale **(**HADS).	The mean Global Health score for the QLQ-C30 was 63.7 Social functioning” scored the highest (mean = 78.1) The worest scores were for Emotional functioning, body image and future perspective (respectively 59.0, 52,9, 52,1) Severe depression and severe anxiety were detected among 8 % and 14 % of study participants, respectively Factors associated: the presence of recurrence since baseline, family history of cancer, low educational status, current social problems, extent of the disease and employment status	-	There is an urgent need for psychosocial support programs and psychological screening and consultation for breast cancer patients at hospitals of the Ministry of Health in Jordan

#### Objectives and study design

In the Thirteen studies, only one study was qualitative [16] and the remaining twelve were quantitative studies [17–28]. Among the quantitative studies, one was an interventional study [21] and eleven were cross-sectional studies [17–19], [22–28].

Two of the studies reported validation or cultural adaptation of QOL questionnaires [18, 27]. Ten articles focused on the impact of breast cancer and its treatment on QOL of women with breast cancer and analyzed the influence of sociodemographic and clinical data [17], [19–26], [28]. The interventional study conducted in Egypt aimed to improve the QOL of affected patients and their families. The intervention was conducted through communication by phone calls, health education messages, restorative (rehabili-tative) therapy for needed cases, social and financial support [21]. The qualitative study conducted in Bahrain explored the experiences, beliefs, perceptions and attitudes of women with breast cancer towards their QOL [16].

#### Questionnaires

The EORTC questionnaires were used in seven studies. Three of them used only the QLQ-C30 [20, 23, 26], four used both The EORTC QLQ – C30 and theEORTCbreast cancer- specific quality of life questionnaire (EORTCQLQ-BR23) [18, 19, 21, 28] and one study used only the specific module for breast cancer the QLQ-BR23 [27]. The studies conducted in Yemen have used the Functional Assessment of Cancer Therapy- Breast (FACT-B) questionnaire to assess the QOL [22, 25]. Other questionnaires were used in two studies conducted in Egypt namely the Breast Impact of Treatment Scale (BITS) [24] and Linear Analogue Self-Assessment Scales (LASA) [17].

#### Global quality of life

Concerning studies using the QLQ-C30, good scores of global QOL were recorded for Arab women living in United Arab Emirates (UAE) and also for Tunisian, Bahraini and Jordanian women with mean score of 74.6,68.5, 63.9, 63.7 respectively [18, 20, 26, 28]. The score obtained for Lebanese [23] was 59.64 while the Kuwaiti women [19] had lower score (45.30).

The first QOL study conducted among Arab women was in Egypt in 1997 has used a LASA and the results were focused on the impact of the modality of treatment on the QOL [17]. The study conducted by Mostafa et al. shown that 38.3 % of Egyptian patients had poor global QOL and 52.8 % had good global QOL [21]. Another study conducted also in Egypt used the BITS and Body Satisfaction scale (BSS) but has not given any scores of QOL [24]. The study conducted in Yemen used FACT-B and reported 77.6 as overall QOL [25].

#### Functioning and Symptoms

According to the EORTC QLQ-C30 and the EORTC BR-23 scores, Arab patients with breast cancer have an average to intense symptoms experience. Within the functional scales, the worst scores were for emotional functioning, body image and future perspective [19, 23, 26, 28].

In Kuwait, findings showed that patients were optimistic about their future health. The best domains of QOL were cognitive, social and sexual functioning. Domains with low scores were general wellbeing and physical functioning. Intense level of symptom experience was hair loss [19]. High scores were reported on functional ability, medical care, spirituality, and relationships for Lebanese women with breast cancer and the most common symptoms were nervousness, sadness, lack of energy and pain [23]. Among Bahraini patients, social functioning scored the highest, whereas emotional functioning and sexual functioning scored the lowest. In addition, the most distressing symptom was fatigue, followed by hair loss as the most intense symptom [26]. Similarly, in Jordan the social functioning scored the highest and emotional functioning, body image and future perspective scored the lowest. Hair loss cased the worst symptom [28].

#### Factors associated with QOL

Studies conducted among Arab women with breast cancer have identified socio-demographic and clinical factors associated with QOL. Results of studies conducted in UAE, Yemen, Libanon and Bahrain have shown strong relationship between clinical factors and QOL. As a result family history of cancer, menopausal status, presence of metastasis, time since diagnosis, symptoms, disease stage, presence of side effects and type of treatment received were significantly associated with QOL [18, 22, 23, 26].

On the other hand, the studies conducted in Yemen, Lebanon and Jordan have highlighted the association of some sciodemographic factors and the QOL of patients[22, 23, 28]. Most factors identified in these studies were monthly income, payments per month for medical expenses andeducational status.

The study conducted in Tunisia showed a significant deterioration in physical, cognitive and social functioning among patients undergoing treatment [20]. In Egypt, the interventional study conducted by Mostapha et al. revealed a significant change in physical, role, emotional, cognitive and social functioning in pre-and post-intervention assessment[21]. A strong relationship was also found among Egyptian patients between QOL and type of treatment, disease stage and presence of secondaries [21].

The results of the two studies conducted in Yemen [22, 25] were different regarding the relationship between age and QOL. The study conducted in 2011 concluded the absence of this relationship [22] while the second conducted in 2012 revealed that Younger women (<50 years) showed significantly lower QOL scores than older women (≥50 years) in all of the QOL subscales [25].

#### Results of the qualitative study

The qualitative study conducted in Bahrain explored the experiences of women who have survived breast cancer and their perception of QOL after diagnosis. Global QOL was expressed by participants in terms of being able to perform every day chores and the ability to function in their daily role as a woman, wife, daughter and employee. The study revealed the important role played by the family and husband in treatment decisions and the importance of spirituality and religion to cope with the disease [16].

## Discussion

The objective of this study was to review published research into QOL among Arab women with breast cancer; analyse the characteristics of these studies and the main results reported. Our review paper focused only on breast cancer and have included both qualitative and quantitative papers.

In Arab countries, breast cancer accounts for 14 % to 42 % of all cancers in women. The physical, functional, psychological and social problems that women live may affect their quality of life. However, the results indicate that research into the QOL of breast cancer patients is poorly developed in Arab countries. After an extensive literature review, we have identified only 13 articles that met inclusion criteria. Despite the general recommendation to include QOL as an outcome in clinical studies involving breast cancer patients [29].

The quantitative studies included in this review focused on the impact of breast cancer and its treatment on QOL and analyzed the influence of socio demographic and clinical data [17, 19–26, 28]. While the qualitative study conducted in Bahrain explored the experiences, beliefs, perceptions and attitudes of women with breast cancer towards their QOL [16].

This review of studies using EORTC C-30 questionnaire shows that the scores of QOL of Arabwomen with breast cancer differ from country to country. Therefore the mean scores of QOL varies from 45.3 to 74.6 on a scale of 100 and good scores were recorded at UAE, Tunisia, Bahrain and Jordan. The lowest mean score was recorded among Kuwaiti Patients (45.3).

Nonetheless, we must be cautious in comparing data from these studies. The literature using the EORTC QLQ-C30 indicates that comparison of data should go beyond the usual presentation of mean scores and significant differences [30–33]. Disparity in QOL scores among Arab women can be related to different interpretation of QOL and selection bias. Indeed the population under the study vary in terms of time since diagnosis, disease stage, treatment received and the questionnaire used might contributed to the observed differences. For instance, studies conducted in Bahrain and Jordan evaluated the QOL of breast cancer survivors, while the researchers in UAE evaluated the QOL of patients under treatment, and studies conducted in Kuwait and Tunisia were carried out among patients under chemotherapy. Moreover, the studies conducted in Yemen and Egypt have used other types of questionnaires and thus the difficulty to compare their results with the other studies remains.

Consistent with other international studies conducted in Australia and Sweden [34, 35], the findings of our review showed that breast cancer and its treatment affect the QOL of patients in physical, psychological and social domains. Among functional scales, high scores were reported onsocial functioning in Kuwaiti, Lebanese, Bahraini and Jordanian patients [19, 23, 26, 28]. Whereas the emotional functioning scored the lowest in Jordanian and Bahraini [26, 28]. Arab patients with breast cancer had average to intense symptoms experience and intense level of symptom experience was hair loss [19, 26, 28].

One of the reasons for having pooremotional functioning may be related to the fact that women with breast cancer have to deal not only with the trauma of disfigurement but also with the fear of rejection by their partners and loss of femininity [36]. They also are subjected to too much pressure because of the burden of working in different fields in addition to the commitments of their roles as mothers and housewives.

The social domain had the highest scores among QOL subscales. This may be referred to effective social support system in the Arab communitieswich plays an important role in reducing the pressure and improving health. The experience of patients is usually influenced by the spiritual and religious context characterized by the solidarity and support. Many studies supported these findings, Hebert et al. indicated that positive religious coping methods predict better mental health and life satisfaction in women with breast cancer [37].

In line with other research on QOL of breast cancer [38–41], the findings of studies conducted among Arab women have shown that clinical and socio demographic factors were associated with QOL. The most clinical factors studied was the type of treatment. The triple modality of treatment predicted the worst QOL [17]. the radiotherapy had significantly less effect compared to chemotherapy [17, 22]. Patients who underwent mastectomy reported lower levels of emotional, cognitive functions, more side effects of treatment and arm symptoms than patients who had undergone lumpectomy [18]. But no difference was found between the patients who underwent mastectomy with reconstruction and those who had mastectomy alone [24].

Similar themes to other studies emerged in terms of impact of socio demographic factors on QOL [39, 40]. The most identified in our review were monthly income, payments per month for medical expenses and educational status. This is supported by many studies which indicated that increased QOL scores were significantly correlated with increased education and better socioeconomics status [22, 38, 40], [42–44]. In Denmark, Peuckmann and his colleagues [45] reported that poor QOL was significantly associated with short education. The same findings were also reported by Esbensen and others [46] who showed that poor economy was associated with low QOL. The family income was significantly influencing the overall QOL as reported by Pandey et al. [47]. The possible justification for this findingsis the ability of the educated women to understand the nature of the disease and to comply with the therapeutic regime more than the uneducated. Moreover, illiterate women with low income are less likely to be screened for breast cancer, would delay seeking care in the presence of symptoms and were diagnosed in later stages of the disease [22].

Although the results of other studies have shown the relationship between age and the QOL of breast cancer patients [39, 48]. Only the study by Sawsan Ba-Khubaira et al. in Yemen has demonstrated this relationship among Arab patients [25]. The findings for the association of age with QOL are conflicting; some correlated poorer QOL with increasing age [49, 50], while others found that younger age patients had more physical wellness and better overall QOL [51]. The inconsistency of these studies may be explained by differences in population background, source of subjects and sample size.

The impact of socio demographic characteristics was far different between Arab countries. This may be explained by economic differences and health policy especially the implementation of early detection and treatment programs of breast cancer.

### Implications for practice and policy

The results highlight the need of a holistic care and the importance of taking into consideration the cultural and religious specificities in the treatment of Arab women with breast cancer. Future research should test intervention to assist Arab women to increase their personal optimism, reduce symptom distress and enhance coping strategies. The findings call for the institution of a psycho-oncology services to address psycho-social outcomes.

### Limitations of this review

Every effort was made to provide a comprehensive and systematic review of the literature. However, it is possible that some studies may not have been captured in the search and screening process and other did not meet inclusion criteria [52–58] (Additional file 1). Also, the difference of the base populations and the questionnaires used to measure QOL does not allow a comparison of QOL between Arab women. It is worth noting that the majority of studies were cross-sectional and had a small sample size and thus the findings could not be generalized to all Arab women with breast cancer.

## Conclusion

This study investigated for the first time published research on QOL among Arab women with breast cancer even though insufficient information is available on QOL in Arab women with breast cancer. Certainly, the reviewer studies were very interesting and provided a working basis for further studies. However, there is a pressing need to support more research and publications to improve QOL of Arab women with breast cancer in Arab countries where the breast cancer incidence rates have increased during recent years.

## Additional file

Additional file 1: Excluded studies and reasons for exclusion. (DOC 55 kb)

## Abbreviations

AIM: African Journals Online and African Index Medicus
ASR: age-djusted standardized incidence rates
BITS: Breast Impact of Treatment Scale
BSS: Body Satisfaction Scale
EORTC: European Organization for Research and Treatment of Cancer
FACT-B: Functional Assessment of Cancer Therapy- Breast
LASA: Linear Analogue Self-Assessment
PRISMA: Preferred Reporting Items for Systematic Reviews and Meta-Analyses
QLQ-BR23: European Organization for Research and Treatment of Cancer breast cancer- specific quality of life questionnaire
QLQ-C30: European Organization for Research and Treatment of Cancer Core quality of life questionnaire
QOL: quality of life
UAE: United Arab Emirates
WHO: World Health Organisation
