# Supplementary material for: Quality of life in Arab women with breast cancer: a review of the literature
Source: Health Qual Life Outcomes. 2016 Apr 27;14:64. doi: 10.1186/s12955-016-0468-9 (PMC4847355; doi:10.1186/s12955-016-0468-9)
Supplement: Additional file 1: — Excluded studies and reasons for exclusion. (DOC 55 kb) [file 12955_2016_468_MOESM1_ESM.doc]

Additional file: Excluded studies and reasons for exclusion

| **Author** | **Study design** | | **Main Focus** | | **Participants** | **Results** | | **Reasons for exclusion** | |  |
| --- | --- | --- | --- | --- | --- | --- | --- | --- | --- | --- |
| Huijer and Abboud [52] | Cross sectional | Descriptive survey targeting adult oncology patients | | 200 Lebanese Patients with Cancer  women with BC account for 44.5% of the study population(89 patients) | | | Predictors of better QOL Lebanese Patients with Cancer : physical, role, and emotional functioning, demographic characteristics, social status  Predictors of poorer QOL Lebanese Patients with Cancer : the total symptoms scale as well as fatigue | | No specific results for patients with breast cancer | |
| Nejjari et al [53] | Cross-cultural adaptation  of the EORTC QLQ-C30 | Evaluation of the psychometric properties  of the Moroccan Arabic version of the EORTC QLQ-C30 | | 125 Moroccan  Patients  16% with breast cancer | | | the Moroccan Arabic version of the  EORTC QLQ-C30 is a reliable and valid measure of the QOL in cancer patients | | No results on the measurement of quality of life of patients with breast cancer | |
| Mnif et al [54] | Cross sectional study | Quality of life of Tunisian women after the treatment of breast  Cancer | | 50 patients in remission at least 3 months after initial  treatment of breast cancer, and 50 women with no history of cancer. | | | Tunisian women had significantly poorer GQOL and functional scales scores in comparison with the general  population | | Abstract in the 19th European Congress of Psychiatry. 2011 | |
| Butow et al [55] | population-based study | Inferior health-related quality of life and psychological well-being  in immigrant cancer survivors | | 596 patients  57 Arab patients including 17 with breast cancer | | | Immigrants with cancer in the  post-treatment survivorship phase have clinically significantly worse QOL and higher reported depression than Anglo-Australian-born cancer survivors matched on cancer diagnosis.  Arabic cancer patients reported the highest depression and worst QOL | | No results on the measurement of the quality of life of Arabic breast cancer patients | |
| Abu obead et al [56] | One group quasi-experimental correlational design | Impact of radiotherapy treatment on Jordanian cancer patients’ quality of life and fatigue | | 82 Jordanian patients who receive radiotherapy as a primary treatment for their cancer  43 patients with breast cancer | | | Significant differences were found between pre- and post- radiotherapy QOL mean total scores  Statistically significant differences were found between pre- and post- radiotherapy fatigue mean total scores  QOL total scores correlated significantly and negatively with total fatigue scores | | No specific findings for patients with breast cancer | |
| Lazenby and Khatib [57] | Cross sectional study | Associations among Patient Characteristics,  Health-Related Quality of Life, and Spiritual Well-Being  among Arab Muslim Cancer Patients | | 159 adult Muslim patients  of which 59 are patients with breast cancer | | | The FACIT-Sp distinguishes between domains of HrQOL and patient characteristics. Further study on the unique contribution of the FACIT-Sp’s Peace and Meaning subscales to HrQOL is needed | | No specific findings for patients with breast cancer | |
| Hoopman et al [58] | Cross sectional study | Translation and validation of the EORTC QLQ-C30 foruse among Turkish and Moroccan ethnic minority  cancer patients in the Netherlands | | Turkish and Moroccan cancer patients in the Netherlands  79 Moroccan patients  18 Breast cancer | | | Data support the use of the QLQ-C30 among Turkish and Moroccan cancer patients residing in the Netherlands. | | No results on the measurement of the quality of life of breast cancer patients | |
